# HMG-CoA reductase promotes protein prenylation and therefore is indispensible for T-cell survival

**DOI:** 10.1038/cddis.2017.221

**Published:** 2017-05-25

**Authors:** Sonja M Lacher, Julia Bruttger, Bettina Kalt, Jean Berthelet, Krishnaraj Rajalingam, Simone Wörtge, Ari Waisman

**Affiliations:** 1 Institute for Molecular Medicine, University Medical Center of the Johannes Gutenberg-University Mainz, Obere Zahlbacher Str. 67, Mainz 55131, Germany; 2 Molecular Signaling Unit, FZI, Institute of Immunology, University Medical Center of the Johannes Gutenberg-University Mainz, Langenbeck Str. 1, Mainz 55131, Germany

## Abstract

Statins are a well-established family of drugs that lower cholesterol levels via the competitive inhibition of the enzyme 3-hydroxy-3-methylglutaryl coenzyme A reductase (HMGCR). In addition, the pleiotropic anti-inflammatory effects of statins on T cells make them attractive as therapeutic drugs in T-cell-driven autoimmune disorders. Since statins do not exclusively target HMGCR and thus might have varying effects on different cell types, we generated a new mouse strain allowing for the tissue-specific deletion of HMGCR. Deletion of HMGCR expression in T cells led to a severe decrease in their numbers with the remaining cells displaying an activated phenotype, with an increased proportion of regulatory T cells (T_regs_) in particular. However, deletion of HMGCR specifically in T_regs_ resulted in severe autoimmunity, suggesting that this enzyme is also essential for the maintenance of T_regs_. We were able to prevent the death of HMGCR-deficient lymphocytes by the addition of either the direct metabolite of HMGCR, namely mevalonate, or the downstream metabolite geranylgeranyl pyrophosphate, which is essential for protein prenylation. However, the addition of cholesterol, which is the final product of the mevalonate pathway, did not inhibit cell death, indicating that protein prenylation rather than the cholesterol biosynthesis pathway is indispensible for T-cell survival.

The 3-hydroxy-3-methylglutaryl coenzyme A (HMG-CoA) reductase (HMGCR) is an endoplasmatic reticulum residing enzyme, which catalyzes the rate-limiting step of cholesterol biosynthesis within the mevalonate pathway.^1^ It catalyzes HMG-CoA conversion to mevalonate and can be competitively inhibited by statins such as lovastatin, pravastatin, mevastatin and simvastatin; or the synthetic statins including fluvastatin, atorvastatin, cerivastatin and rosuvastatin.^2^ These statins differ in their half-life potency and lipophilicity and are widely used as drugs to lower the cholesterol level of patients with cardiovascular disease.^3^

In recent years, it became evident that statins also have pleiotropic immunological effects^4, 5, 6^ and can even prevent tumor development.^7, 8^ When used to treat mice in a model of multiple sclerosis, statins were shown to ameliorate the disease.^6^ Disease reduction was attributed to reduced proliferation of the self-reactive T cells and a shift from pro-inflammatory interferon-*γ* (IFNγ) producing T_H_1 cells to anti-inflammatory IL-4 producing T_H_2 cells and a subsequent decrease in inflammation of the central nervous system.

These effects of statin treatment are most likely not mediated by decreased cholesterol levels, but rather could be due to decreased protein prenylation, another HMGCR-dependent reaction. Protein prenylation is a posttranslational modification of proteins, which results in the covalent connection of these proteins with the mevalonate pathway intermediates farnesyl pyrophosphate or geranylgeranyl pyrophosphate (GGPP).^2^ The lipophilic prenyl groups enable proteins to anchor to cell membranes or facilitate protein–protein interactions. Some important prenylated proteins include members of the Ras superfamily of small GTPases, such as Ras and Rho, involved in proliferation and differentiation processes of cells.^2^ To better understand the role of statins in autoimmunity and elucidate their effects on HMGCR and other putative targets, we generated a new mouse strain that enables tissue-specific deletion of HMGCR via Cre/loxP system. These mice were crossed to the CD4-cre mice, resulting in deletion of HMGCR in all *αβ* T cells. We could show that HMGCR deletion in T cells leads to their death, which could be rescued by the external addition of mevalonolactone or GGPP, but not cholesterol. Our data demonstrate that HMGCR is indispensible for the survival of T cells via the protein prenylation pathway.

## Results

### HMGCR deletion in T cells leads to a dramatic reduction in their cell numbers and an enrichment of activated T cells

**Figure 1:**
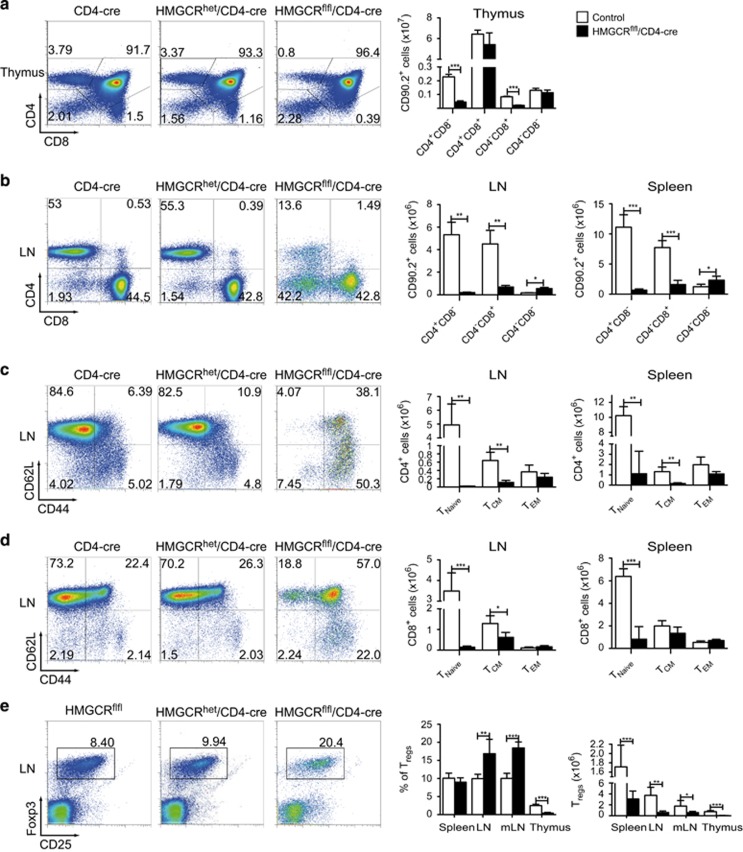
HMGCR deletion in T cells results in a dramatic reduction in their numbers. Cells were analyzed by FACS and pregated on CD90.2^+^ cells (**a** and **b**) and then further gated for CD4^+^ (**c**), CD8^+^ (**d**) or CD4^+^FoxP3^+^ T cells (**e**). T_Naive_: CD62L^+^CD44^−^, T_CM_: CD62L^+^CD44^+^ and T_EM_: CD44^+^CD62L^−^. HMGCR^het^/CD4-cre animals were used as controls (*n*≥3±S.D.). All significant differences are shown and marked with stars "*", "**" and "***"

The gene *Hmgcr* is located on chromosome 13 in mice and consists of 20 exons, which can be expressed as 11 different splice variants, 7 of which are protein coding. For the generation of a conditional knockout mouse, we opted to flank exon 15, which codes for an essential part of the catalytic domain of HMGCR, with loxP sites. Deletion of exon 15 should lead to a downstream frame shift of exons 16–20, resulting in an inactive enzyme (see details in Supplementary Figure 1). To evaluate the importance of HMGCR for T cells, we crossed HMGCR^flfl^ mice to CD4-cre animals, resulting in HMGCR^flfl^/CD4-cre mice where this gene was inactivated in all *αβ* T cells. As seen in Figure 1a, deletion of HMGCR, starting during the double positive (DP) stage in the thymus, resulted in a significant reduction in both CD4^+^ as well as CD8^+^ single positive (SP) thymocytes. The reduction of CD4^+^ and CD8^+^ T cells was even more evident in the lymph nodes (LN) and spleen of the mutated mice, as seen in Figure 1b. Most of the remaining CD4^+^ and CD8^+^ T cells in these mice showed an activated phenotype (Figures 1c and d). Interestingly, we noticed an increase in the percentage, but not total number, of regulatory T cells (T_reg_) within the CD4^+^ population (Figure 1e). The deletion of just one *HMGCR* allele (HMGCR^het^) did not affect the cells of the immune system in steady state (Figures 1a–e). However, as HMGCR^flfl^/CD4-cre mice barely have T cells, we were wondering whether *HMGCR* deletion in only one allele would cause a dose-dependent effect after T-cell activation *in vivo*. Since it was previously shown that treatment with HMGCR inhibitors (statins)^6^ resulted in amelioration of disease in the EAE model of multiple sclerosis, we induced EAE in the HMGCR^het^/CD4-cre mice. Mice were scored daily according to signs of paralysis and weight loss (Supplementary Figures 2a and b). However, we detected no difference in disease development when compared with the control animals (see also Supplementary Figure 2c), suggesting sufficient HMGCR expression from one allele.

### The majority of the T cells in HMGCR-deficient animals consist of T cells that escaped Cre-mediated deletion

**Figure 2:**
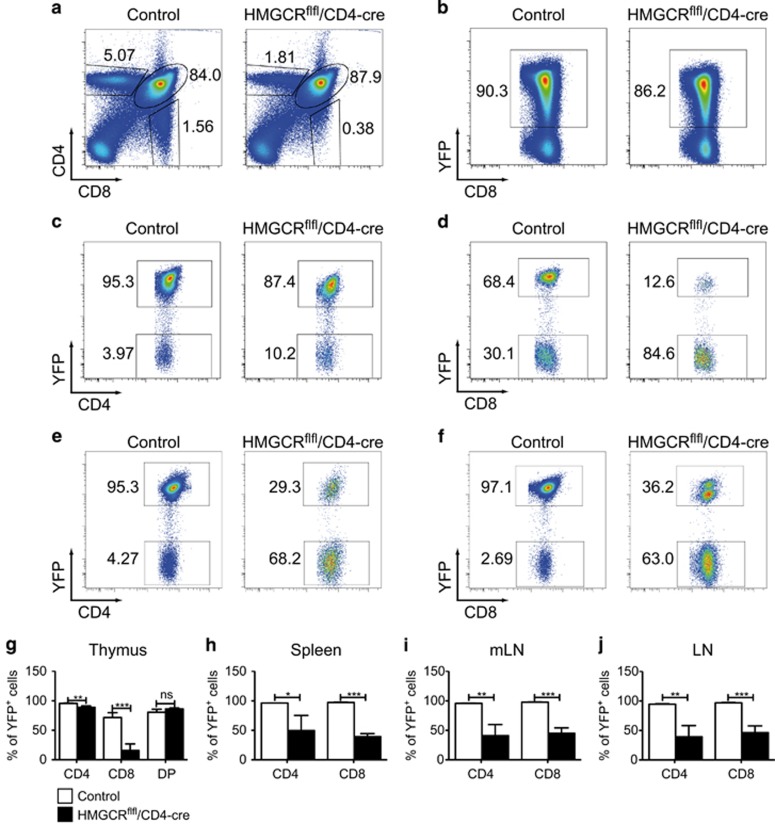
The majority of the remaining mature T cells in the T-cell-specific HMGCR-deficient mice are Cre-LoxP escapees. HMGCR^flfl^/CD4-cre and HMGCR^het^/CD4-cre mice (controls) were crossed to the YFP-reporter mice. The FACS plots represent cells from the thymus (**a**–**d**) or LN (**e** and **f**). Thymocytes (**a**) were gated by expression of CD4^+^CD8^+^ (DP, **b**), CD4^+^ (**c**) or CD8^+^ (**d**); lymphocytes were gated for TCR*β*^+^CD4^+^ (**e**) or TCR*β*^+^CD8^+^ (**f**). Statistics in **g**–**j** are shown for the indicated tissues (*n*≥3±S.D.). The experiment is representative of two independent experiments

As HMGCR^flfl^/CD4-cre mice showed a dramatic reduction in T-cell numbers, we were wondering if the remaining cells had escaped Cre recombination, indicating that the deletion of HMGCR leads to obligatory cell death. To detect possible ‘escapees,’ we crossed YFP-reporter mice to the HMGCR^flfl^/CD4-cre animals resulting in mice where HMGCR-deficient T cells express YFP. As controls, we used mice deficient for only one allele of *HMGCR*. This system allowed us to track HMGCR-deleted cells, as they would express YFP while Cre-escaped cells should not. For analysis of thymocytes, we gated on DP, CD4 SP cells or CD8 SP cells (Figure 2a) and investigated YFP^+^ and YFP^−^ cells (Figures 2b–d). In the DP state, the knockout and control mice showed comparable frequencies of YFP^+^ cells (Figures 2b and g). Interestingly, we observed only a mild decrease in YFP^+^ CD4^+^ SP cells in the thymus (Figures 2c and g), but at the same time there was a strong shift to YFP^−^ T cells among the CD8^+^ SP cells in HMGCR^flfl^/CD4-cre animals (Figures 2d and g). As the control CD8^+^ SP population also contains 30% of YFP^−^ cells, it is likely that this population includes CD8^low^ T cells that were pre-DP and therefore did not yet go through Cre-mediated recombination. In contrast to the thymus, we found that about two thirds of all CD4^+^ and CD8^+^ T cells in the peripheral immune organs of HMGCR^flfl^/CD4-cre mice, including the spleen, LN and mesenteric LN expressed no YFP and therefore were Cre escapees (Figures 2e–f and h–j).

Next, we analyzed whether there was any preference in YFP expression among different T-cell subsets, by making use of cell surface markers that distinguish naive T cells, memory T cells (Supplementary Figures 3a and b) and T_regs_ (Supplementary Figures 3c and d). The YFP^+^ knockout T cells displayed a strongly activated phenotype and an increase in percentage of T_regs_, as shown in Figures 1c–e, with comparable results. However, similarly to the memory/activated T cells, the total number of the T_reg_ cells was significantly reduced (Supplementary Figure 3d). Thus, deletion of HMGCR may give a relative advantage to developing an activated T-cell phenotype. However, these cells also need a functional *HMGCR* gene to survive.

### HMGCR is essential for the survival of both T and B cells

**Figure 3:**
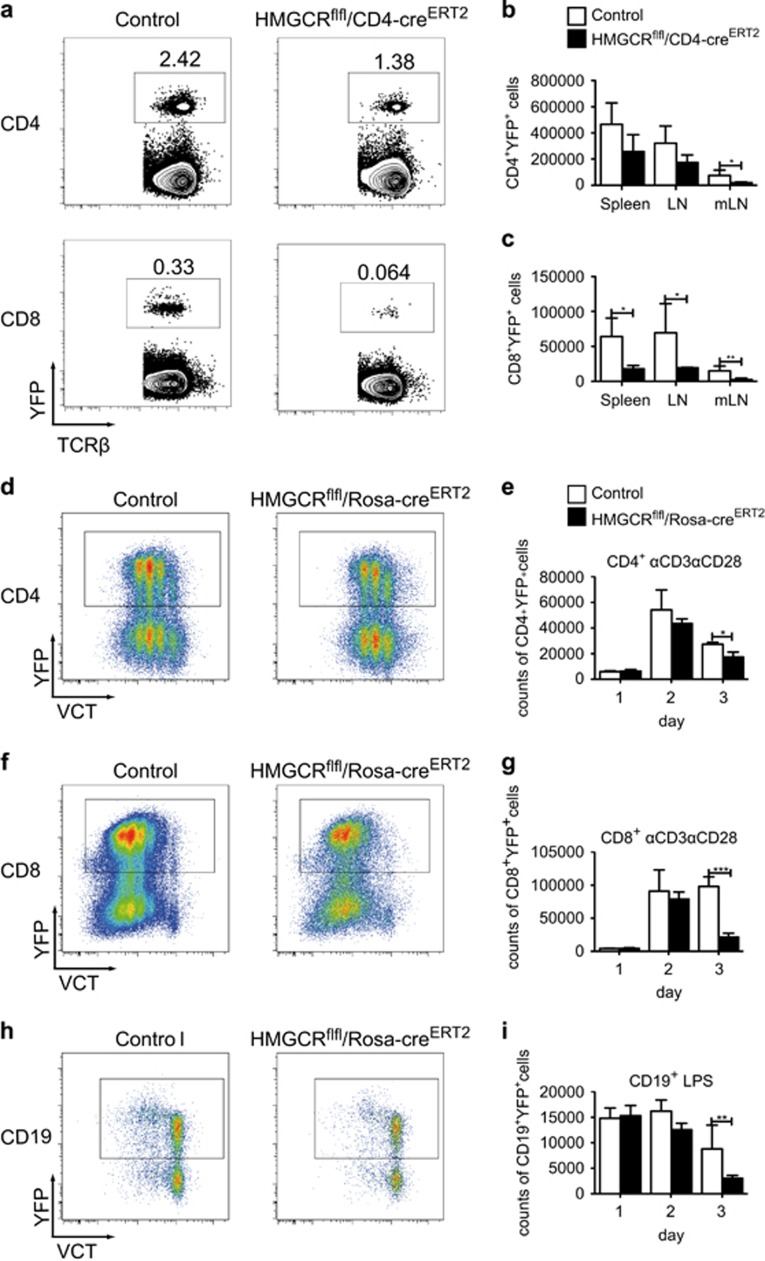
HMGCR is essential for the survival of T and B cells. HMGCR^flfl^/CD4-cre^ERT2^ as well as control mice (HMGCR^het^/CD4-cre^ERT2^) were crossed to the YFP-reporter mice and analyzed 4 weeks after TAM injection. Shown are percentages of T cells in mesenteric LN (**a**) and total cell numbers in spleen, LN and mesenteric LN (**b** and **c**) (*n*≥3±S.D.). VCT-labeled splenocytes of HMGCR^flfl^/Rosa-cre^ERT2^ as well as control mice (HMGCR^het^/Rosa-cre^ERT2^) including the YFP-reporter were cultured under T-cell conditions (**d**–**g**) or under B-cell conditions (**h** and **i**) and 1*μ*M 4-OH TAM for the indicated times and subsequently analyzed for cell numbers of YFP^+^CD4^+^ T cells (**d** and **e**), YFP^+^CD8^+^ T cells (**f** and **g**) and YFP^+^CD19^+^ B cells (**h** and **i**) (*n*≥3±S.D.). The experiment is representative for two independent experiments. All significant differences are shown and marked with stars "*", "**" and "***"

To analyze the mechanism of T-cell death *in vivo*, we crossed the HMGCR^flfl^/YFP^flfl^ mice to the CD4-Cre^ERT2^ line. In these mice, activation of the Cre recombinase is induced on tamoxifen (TAM) injection, resulting in the simultaneous deletion of HMGCR and expression of YFP specifically in CD4^+^ T cells.^9^ The use of a YFP-reporter in this inducible Cre mouse line is important, since efficiency of deletion may be low and one can specifically track these cells and compare them to cells where HMGCR was not deleted, in the same mice. We injected TAM on 4 consecutive days, and 4 weeks later we noticed a mild reduction in the percentage (Figure 3a) and the number of CD4^+^YFP^+^ cells in the mesenteric LNs (Figure 3b). In the spleen and peripheral LNs, we also noticed a tendential reduction in CD4 T-cell numbers (Figure 3b). In contrast, we noted a dramatic reduction in both the percentage (Figure 3a) and total number of the CD8^+^YFP^+^ cells in all analyzed organs (Figure 3c), indicating that CD8^+^ T cells are more sensitive to cell death after HMGCR deletion than CD4^+^ T cells.

To further investigate the survival ability of cells after HMGCR deletion, we crossed the HMGCR^flfl^YFP^flfl^ mice to the Rosa-cre^ERT2^ line. In these mice, the Cre is active in all cell types following the treatment with TAM. This should allow us to also investigate other cell types. Moreover, this strain offers better deletion efficiency in the CD4 T-cell compartment compared with the CD4-cre^ERT2^ mice. At first, we isolated splenocytes from HMGCR^flfl^/Rosa-cre^ERT2^ mice and from HMGCR^het^/Rosa-cre^ERT2^ control animals and cultured them with 4-OH TAM to induce the HMGCR deletion under T-cell activating conditions (1 *μ*g/ml *α*CD3, 6 ng/ml *α*CD28 treatment). To rule out that a reduction in cell numbers might be due to reduced proliferation, we labeled cells with CellTrace Violet stain (VCT). Cells were analyzed by flow cytometry after 1–3 days in culture. As displayed in Figure 3, we noticed a significant reduction of both CD4^+^ (Figures 3d and e) as well as CD8^+^ T-cell numbers (Figures 3f and g) 3 days after culture. Importantly, such differences were not present if the cells were not activated with *α*CD3 and *α*CD28 (data not shown). To test if the reduced cell numbers of T cells we observed are due to apoptotic cell death, we analyzed the numbers of CD4^+^ and CD8^+^ T cells 3 days after treatment with *α*CD3 and *α*CD28 for early (AnnexinV^+^7AAD^−^) and late (AnnexinV^+^7AAD^+^) apoptosis. Indeed, we could detect an increased late apoptotic population in HMGCR-deficient CD4^+^ (Supplementary Figure 4a) and CD8^+^ (Supplementary Figure 4b) T cells. In addition, cell survival was partially rescued by the pan-caspase inhibitor Q-VD-OPh, suggesting that caspase-mediated cell death is at least partially the mechanism of cell death on HMGCR deletion.

As the ROSA26-driven Cre also allows for deletion in other cells, we also analyzed B cells in these cultures. As seen in Figures 3h and i, we could show that also B cells, when activated with LPS, cannot properly survive in culture when HMGCR is deleted. Similarly to T cells, we could see no difference between control and HMGCR-deleted cultures if the B cells were not activated (data not shown).

### HMGCR deletion in T_regs_ causes a scurfy-like phenotype

**Figure 4:**
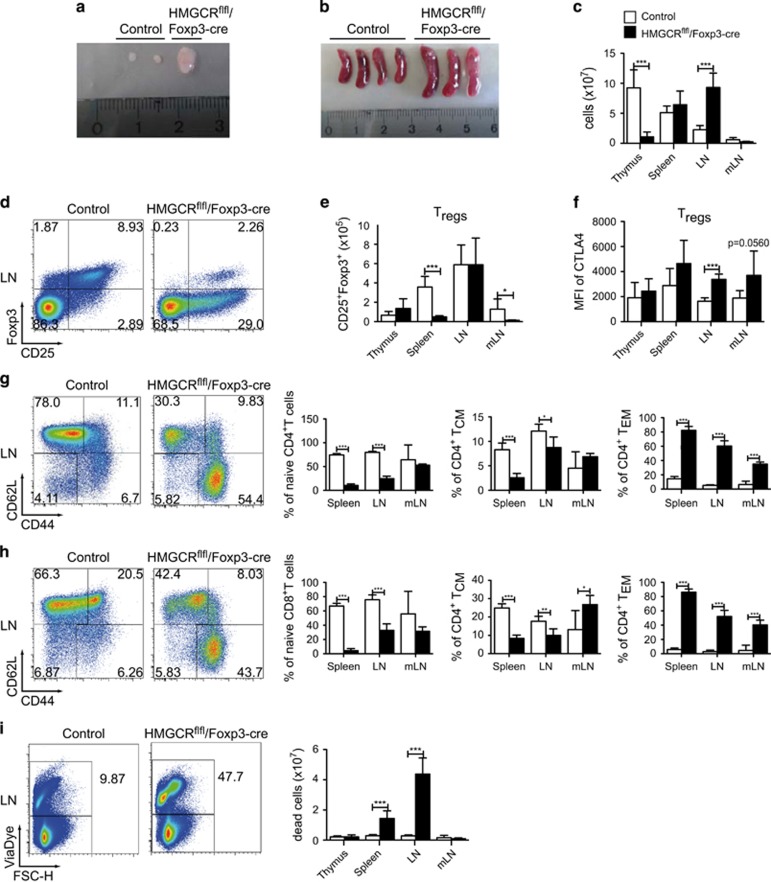
T_reg_-specific HMGCR deficiency leads to a scurfy-like disease in the mice. Shown are the LNs (**a**), spleens (**b**) and absolute cell numbers (**c**) of HMGCR^flfl^/Foxp3-cre and control littermates at the age of 3.5 weeks. T cells were pregated for CD90^+^CD4^+^ and further analyzed for the indicated markers (**d** and **e**) or further gated for the expression of FoxP3 and analyzed for CTLA4 expression (**f**). In addition, for the same mice, are shown naive, T_CM_ and T_EM_ cells of CD4^+^ (**g**) and CD8^+^ (**h**) T-cell populations; Furthermore, we analyzed the percentage and numbers of dead cells (**i**); controls are: WT, Foxp3^+/Y^, HMGCR^het^/Foxp3-cre^+/−^, 2 HMGCR^het^/cre^+/+^, HMGCR^flfl^/Foxp3^+/−^; knockout HMGCR^flfl^/Foxp3-cre mice are HMGCR^flfl^/Foxp3^+/Y^ or HMGCR^flfl^/Foxp3^+/+^ (*n*≥5±S.D.). All significant differences are shown and marked with stars "*", "**" and "***"

Since we observed an increase in the percentage of T_regs_ within the CD4^+^ T-cell population in HMGCR^flfl^/CD4-cre mice, we were wondering if HMGCR is less vital for T_regs_ compared with other T-cell populations. To address this, we crossed the HMGCR^flfl^ mice to the Foxp3-IRES-cre, resulting in mice with specific deletion of HMGCR in regulatory T cells. Interestingly, these mice were smaller in size (data not shown), and had larger LNs (Figures 4a and c) and spleen (Figures 4b and c) compared with littermate controls. Because of the extreme phenotype, which resembles that of scurfy mice lacking T_regs_,^10^ we analyzed these mice during early ontogenesis, already at the age of 3.5 weeks. Importantly, the proportion of T_regs_ was dramatically reduced compared with littermate controls (Figures 4d and e). However, due to increased LN size this was not true for absolute cell numbers in LNs. In addition, the remaining T_regs_ showed increased CTLA4 expression in the LN (Figure 4f), possibly to suppress the ongoing inflammation. Another feature shared with scurfy mice is the complete disappearance of the DP population in the thymus (Supplementary Figure 5a) and increased numbers of CD4^+^ and CD8^+^ cells (Supplementary Figure 5b) in the LN. We further detected a clear shift from the naive to the T_EM_ cell population among CD4^+^ (Figure 4g) and CD8^+^ (Figure 4h) cells of the mutant mice, compared with the control animals. In agreement with the general activation state of the immune system of these scurfy-like animals, we also observed a dramatic increase in the percentage and absolute cell number of IL-17A^+^ (T_H_17) and IFN*γ*^+^ (T_H_1) T cells (Supplementary Figure 5c). In addition, the number of IFN*γ*-secreting CD8^+^ T cells was increased in mice that had lost HMGCR expression in regulatory T cells (Supplementary Figure 5d). The inflammatory phenotype was accompanied with increased cell death in spleen and LN (Figure 4i). Overall, our data suggest that HMGCR is also essential for the survival of regulatory T cells in addition to that of the conventional T cells.

### The cell death caused by HMGCR deletion can be rescued by mevalonolactone or GGPP application

**Figure 5:**
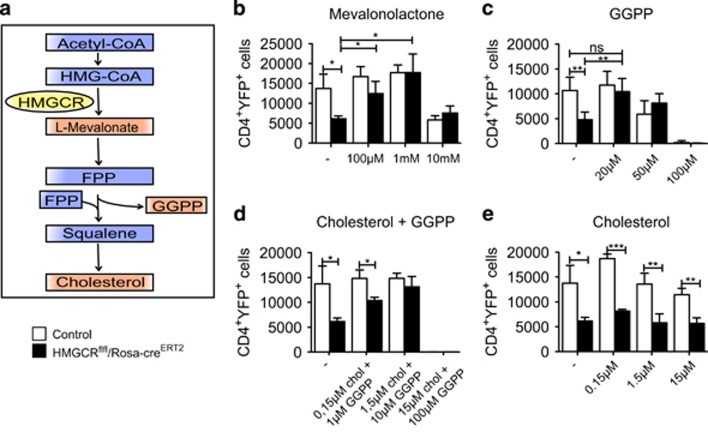
Cell death followed by HMGCR deletion can be rescued by addition of mevalonate or GGPP. (**a**) Schematic representation of the mevalonate pathway. VCT-labeled splenocytes of HMGCR^flfl^/Rosa-cre^ERT2^ as well as control mice (HMGCR^het^/Rosa-cre^ERT2^) including YFP reporter were cultured under T-cell conditions and 1 *μ*M 4-OH TAM together with mevalonolactone (**b**), GGPP (**c**), cholesterol and GGPP (**d**) or cholesterol alone (**e**) and gated for YFP and CD4 expression (*n*=3±S.D.). The experiment is representative of two independent experiments

We next wanted to investigate the mechanism by which HMGCR deficiency causes cell death. To this end, we opted to add downstream components of the mevalonate pathway during *in vitro* T-cell activation and potentially rescue the observed cell death (Figure 5a). To achieve that, we cultured splenocytes isolated from HMGCR^flfl^/Rosa-cre^ERT2^ animals together with 4-OH TAM and *α*CD3*α*CD28. In addition, either the direct metabolite of HMGCR catabolism, named mevalonate, or the more downstream metabolites cholesterol and GGPP were added alone or in combination. We used (±)-mevalonolactone instead of L-mevalonate, since it has better uptake rates by cells and can be easily converted to L-mevalonate. The cell death after HMGCR deletion was rescued in CD4^+^ T cells treated with mevalonolactone (Figure 5b) as well as with GGPP alone (Figure 5c) or with cholesterol and GGPP together (Figure 5d) but not with cholesterol alone (Figure 5e). Altogether, GGPP, which is important for protein prenylation, seems to be important for the survival of CD4^+^ T cells.

## Discussion

Using a novel mouse strain that allows us to conditionally delete HMGCR, we found that HMGCR is critically important for the survival of T cells. The reduced cell number observed *in vivo* could be explained by increased apoptosis in HMGCR-deleted CD4^+^ and CD8^+^ T cells. This confirms previous publications showing increased cell death in PBMCs after the application of high amounts of statins.^11^ However, as statins do not exclusively target HMGCR and T cells, we could demonstrate for the first time that HMGCR deletion in T cells causes their death *in vitro* and *in vivo.* Moreover, we could show that the majority of HMGCR-deficient T cells die via caspase-dependent apoptosis. Unlike prior reports^12, 13^ which demonstrated decreased proliferation of lymphocytes and other cell types after statin treatment, the decreased numbers of T cells and B cells in our experiments were not due to reduced proliferation rates. However, activation of B and T cells was necessary to cause cell death in our experiments suggesting that cell activation accelerates cell death due to an increased need of the mevalonate pathway metabolites. Interestingly, HMGCR was recently shown as a potential target for cancer treatment.^7, 8, 14^ Indeed, it was demonstrated that highly proliferative cells were killed more efficiently with lower statin doses, as compared with non-proliferating cells.^11^ Furthermore, a recent study showed reduced mortality in patients with lung, bowel, breast or prostate cancer who were also diagnosed with hyperlipidaemia.^15^ The authors suggest that medication, such as statins, led to this outcome. Thus, it is of interest to explore a possible link between statin application and cancer development.

We could rescue the cell death seen after HMGCR deletion *in vitro* by adding the direct downstream product of HMGCR, namely mevalonate. Furthermore, to determine whether HMGCR is essential for cholesterol biosynthesis, protein prenylation or for both pathways, we added cholesterol and/or GGPP to the cultured cells. We found that GGPP was needed for the rescue of cell death, while cholesterol alone showed no impact. However, it needs to be mentioned that our culture media contains 10% fetal bovine serum, which might also include cholesterol in low concentration.

Since HMGCR is the key enzyme, not only in cholesterol biosynthesis, but also for the production of small metabolites needed for protein prenylation, we assume that the deletion of HMGCR in any other cell type will also cause cell death. Indeed, we could also induce cell death in B cells via HMGCR deletion. Nevertheless, it may be interesting to test the necessity of this metabolic pathway in other cells types, using the appropriate Cre-driver lines.

When we compared CD4^+^ with CD8^+^ T cells, we noticed that CD4^+^ T cells survived better than the latter after HMGCR deletion: The CD8^+^ SP population exhibited a more severe reduction compared with the DP and CD4^+^ SP T-cell population of the thymus. In addition, the HMGCR deletion in HMGCR^flfl^/CD4-cre^ERT2^ mice demonstrated that CD8^+^ T cells might be more sensitive to cell death compared with CD4^+^ T cells. The difference in survival between CD4^+^ and CD8^+^ T cells may be due to the different developmental stages during which most of these cells lost the expression of HMGCR. Although recombination in the CD8-expressing T cells can only occur during the DP stage in the thymus, CD4-expressing cells are subjected to Cre-mediated recombination at every stage of their development. As the majority of the CD4-expressing cells are naive peripheral T cells, this is the main population affected by the TAM treatment. The observed difference between CD4 and CD8 T cells suggests that when cells develop and proliferate they require HMGCR expression, while the resting cells might survive without it, at least for the period of time tested.

Closer analysis of the remaining activated T cells in the HMGCR^flfl^/CD4-cre mice showed an increased T_reg_ population within the CD4^+^ T-cell compartment. On the one hand, this might be a result of the dramatic cell death, which initiates a cytokine storm with subsequent increase in the activation of T cells. On the other hand, HMGCR-deficient T_regs_ might have a differentiation advantage after HMGCR deletion. It was also shown that murine^16^ and human T cells^17^ can better differentiate to T_regs_ after the application of certain statins, such as atorvastatin, but not mevastatin or pravastatin, and show increased suppressive capacity.^17, 18^ To elucidate whether the increased proportion of the T_reg_ population is the outcome of increased cell death or a differentiation advantage, we crossed the Foxp3-IRES-cre mice to the HMGCR^flfl^ mice. Offspring of these mice showed a scurfy-like phenotype with reduced T_reg_ numbers, dramatically increased T_eff_ cell populations and massive cell death. The remaining T_regs_ could not revert the phenotype even with increased expression of suppressive receptors, such as CTLA4. Thus, even if statin treatment leads to increased T_reg_ differentiation and suppressive capacity, a full deletion of HMGCR expression also causes death of T_reg_ cells.

Statins have already been used for a long time to treat patients with cardiovascular disease and their use also showed decreased disease in the T-cell-driven disease EAE^6^ – the mouse model of multiple sclerosis. To explore this issue, we induced EAE in mice deficient for one allele of *Hmgcr* and detected no effect of such heterozygous deletion. Also of worth to mention, the statins applied in therapies for MS patients either alone or with IFN*β* also did not exhibit beneficial effects thus far.^19^ On the other hand, statin application showed promising effects in the treatment of other neurodegenerative disease,^20^ such as cerebrovascular disease,^21^ Parkinson’s disease^22^ and Alzheimer’s disease.^23^ To reach an optimal neuroprotective effect of statins in patients, as well as in mice, a good diffusion of statins through the blood–brain–barrier is necessary, which is highly dependent on the lipophilicity of the applied statin.^24, 25, 26, 27^ Importantly, for the induction of EAE, pertussis toxin is injected; a drug that can lead to blood–brain–barrier disruption.^28^ The application of pertussis toxin might therefore enable better entry of statins to the central nervous system, which may contribute to the amelioration of EAE. The experiments reported here suggest that statins have other targets besides T cells that are responsible for the ameliorated disease previously reported.^6^ However, it might be that the deletion of only one allele of *HMGCR* is not sufficient to be able to elucidate the role of HMGCR in T cells.

Altogether, we could show that HMGCR is essential for the survival of T cells. Cell death could be inhibited only by either GGPP, which is important for protein prenylation, or by mevalonolactone, which is upstream of the GGPP and cholesterol biosynthesis, and the direct metabolite of HMGCR. However, cholesterol alone could not rescue cell death, indicating that protein prenylation rather than the cholesterol biosynthesis pathway is indispensible for T-cell survival.

## Material and methods

### Mice

HMGCR mice were generated as described in the results. To this mouse strain we crossed the YFP^flfl^ reporter mouse^29^ and the following Cre lines: CD4-cre,^30^ CD4-cre^ERT2^ (ref. 9), Rosa-cre^ERT2^ (ref. 31) (made available to our group by Dr. Ernesto Bockamp, Mainz) and Foxp3-IRES-cre.^32^ All strains were kept on the C57BL/6 background. All experiments were carried out under the terms of the guidelines of the Central Animal Facility Institution of Mainz and in agreement with relevant laws and guidelines with permission by the state Rhineland-Palatinate.

### Organ preparation

Single cell suspensions were obtained via passing the organs through a 40 *μ*M sterile sieve in Dulbecco’s phosphate buffered saline with 2% FCS (FACS buffer). Red blood cells of the spleen were lysed by a 2 min incubation of the cells in Tris-ammonium chloride, pH 7.2.

### TAM injection

TAM (TAM, Sigma) was suspended at 37 °C for 60 min in olive oil (MP Biomedicals). A measure of 2 mg TAM was administered i.p. on 4 consecutive days.

### Cell culture

Single cell suspensions of splenocytes were labeled with CellTrace Violet stain according to the manufacturer’s instructions (Life Technologies and concentrated to 4 × 10^5^–5 × 10^5^ cells in 96 flat-well plates in 200 *μ*l T cell media (RPMI with 10% FCS, 10 mM HEPES, 50 mM 2-mercaptoethanol, 2 mM L-Gln, 1% non-essential amino acids (MEM), 100 units/ml penicillin, 100 mg/ml streptomycin and 1 mM sodium pyruvate) or B-cell media (same as T-cell media but DMEM instead of RPMI). All conditions contained 1 *μ*M 4-OH TAM to induce the HMGCR deletion *in vitro*. For T-cell stimulation, the conditions contained 1 *μ*g/ml *α*CD3, and 6 ng/ml *α*CD28 (BioXCell); for B-cell stimulation 20 *μ*g/ml LPS (Sigma) was added. In some conditions (±)-mevalonolactone (Sigma), a cell-permeable version of GGPP-geranylgeraniol (Sigma), Q-VD-OPh (Sigma) and NBD-6 Cholesterol (Avanti) was used. The cultured cells were analyzed on days 1–3 via FACS analysis.

### Flow cytometry

Single cell suspensions were blocked with Fc-Block (BioXCell) in FACS buffer and surface stained with the following monoclonal antibodies: CD4 (Biolegend, RM4-5), CD8 (eBioscience, 53-6.7), CD19 (6D5, Biolegend), CD25 (BD, PC61), CD44 (eBioscience, IM7), CD62L (MEL-14, eBioscience), CD90.2 (eBioscience, 53–2.1), *γδ*TCR (eBioscience, eBioGL3), Gr-1 (BD, RB6-8C5), F4/80 (eBioscience, BM8). To exclude dead cells, cells were also stained with the fixable viability dye ef780 (eBioscience).

For T_reg_ stainings, cells were fixed and permeabilized according to the PE anti-mouse/rat Foxp3 staining set (eBioscience) and intracellularly stained for Foxp3 (eBioscience, FJK-16s) and CTLA4 (BD, UC10-4F10-11). The cells were acquired with the FACS Canto II (BD Pharmingen) and analyzed with FlowJo Version 8.87. Cells were usually pregated for a lymphocyte gate, singlets and living cells.

### Induction of EAE and clinical assessment

Mice were immunized subcutaneously at the base of the tail with 50 *μ*g MOG_35–55_ peptide (Gene Script) emulsified in complete Freund’s adjuvant (Difco Laboratories, Detroit, MI, USA) supplemented with 1.1 mg of heat‐inactivated *Mycobacterium tuberculosis* (Difco). 200 ng of pertussis toxin (List Biological Laboratories) was intraperitoneally injected on the same day and a second dose 2 days later. Mice were scored each day for clinical signs of EAE as previously described^33^ with modifications. The clinical assessment scale was graded from 0 to 6 as follows: 0, no disease; 0.5, partial loss of tail tonicity; 1, complete loss of tail tonicity; 1.5, partially impaired righting reflex on attempt to roll over (within 3 s); 2, impaired righting reflex; 2.5, partial hindlimb paresis resulting in staggering gait; 3, one hindlimb fully paralysed; 4 full hindlimb paralysis; 4.5, starting forelimb paresis; 5, forelimb paresis resulted in inability to move the body; 5.5, moribund; and 6, dead animal.

### Statistical analysis

Where appropriate, all differences were evaluated with a two-tailed Student’s *t*-test, using Prism (GraphPad 5 Software Inc.). Data are presented as mean±S.D. A *P*-value of 0.05 or less was considered significant (*P<0.05, **P<0.005, ***P<0.001).
