# Supplementary material for: HMG-CoA reductase promotes protein prenylation and therefore is indispensible for T-cell survival
Source: Cell Death Dis. 2017 May 25;8(5):e2824–. doi: 10.1038/cddis.2017.221 (PMC5520735; doi:10.1038/cddis.2017.221)
Supplement: Supplementary Figures [file cddis2017221x1.pdf]

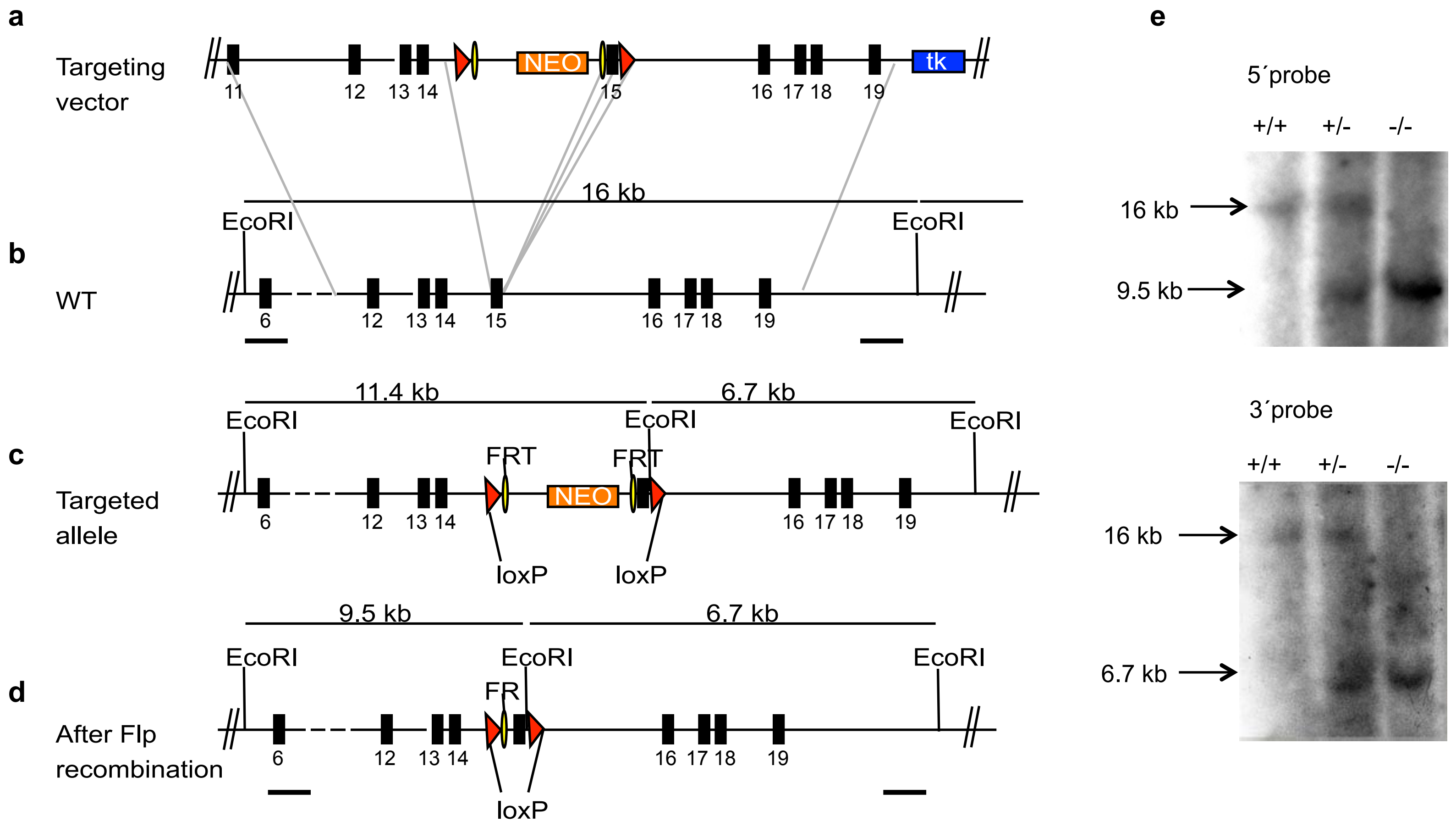

Supplementary Figure 1

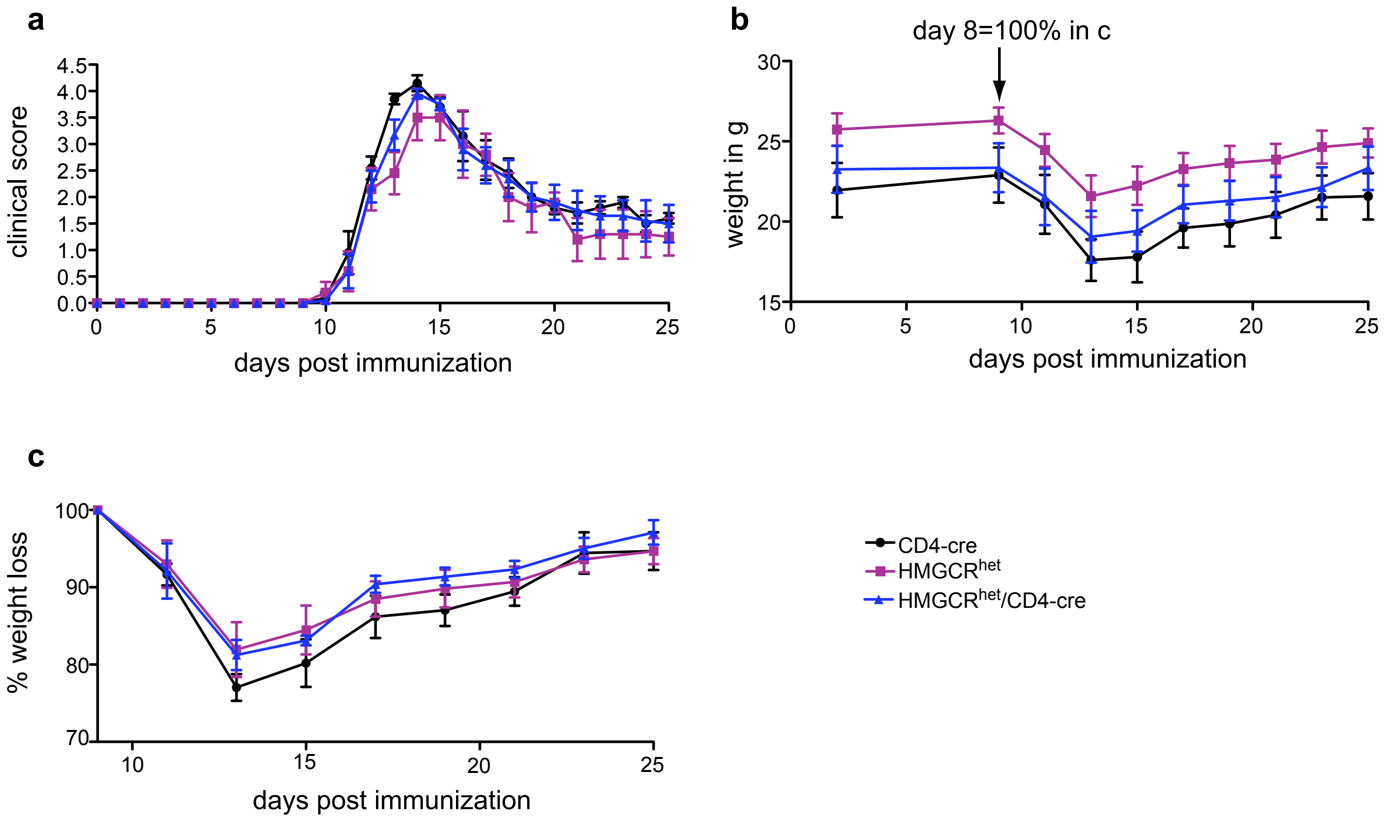

Supplementary Figure 2

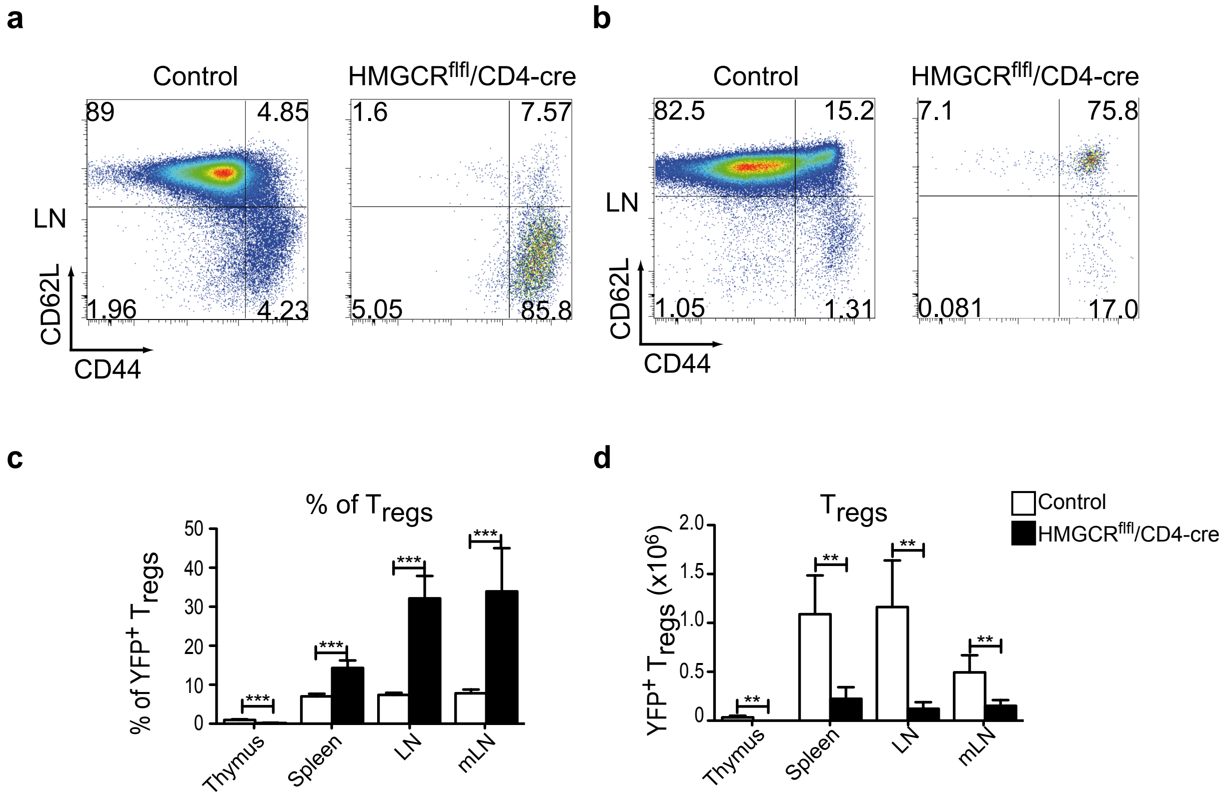

Supplementary Figure 3

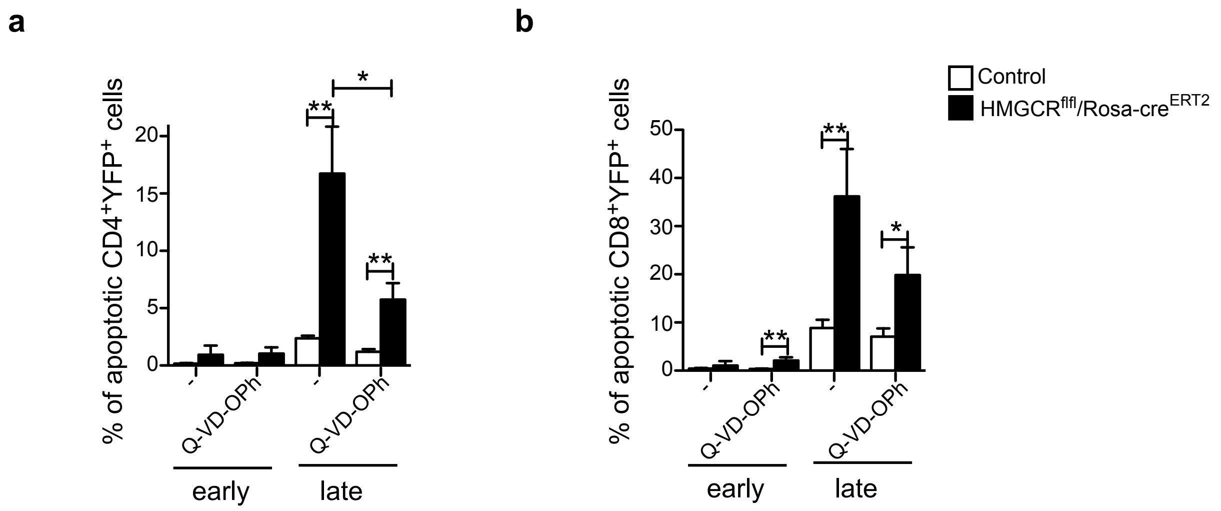

Supplementary Figure 4

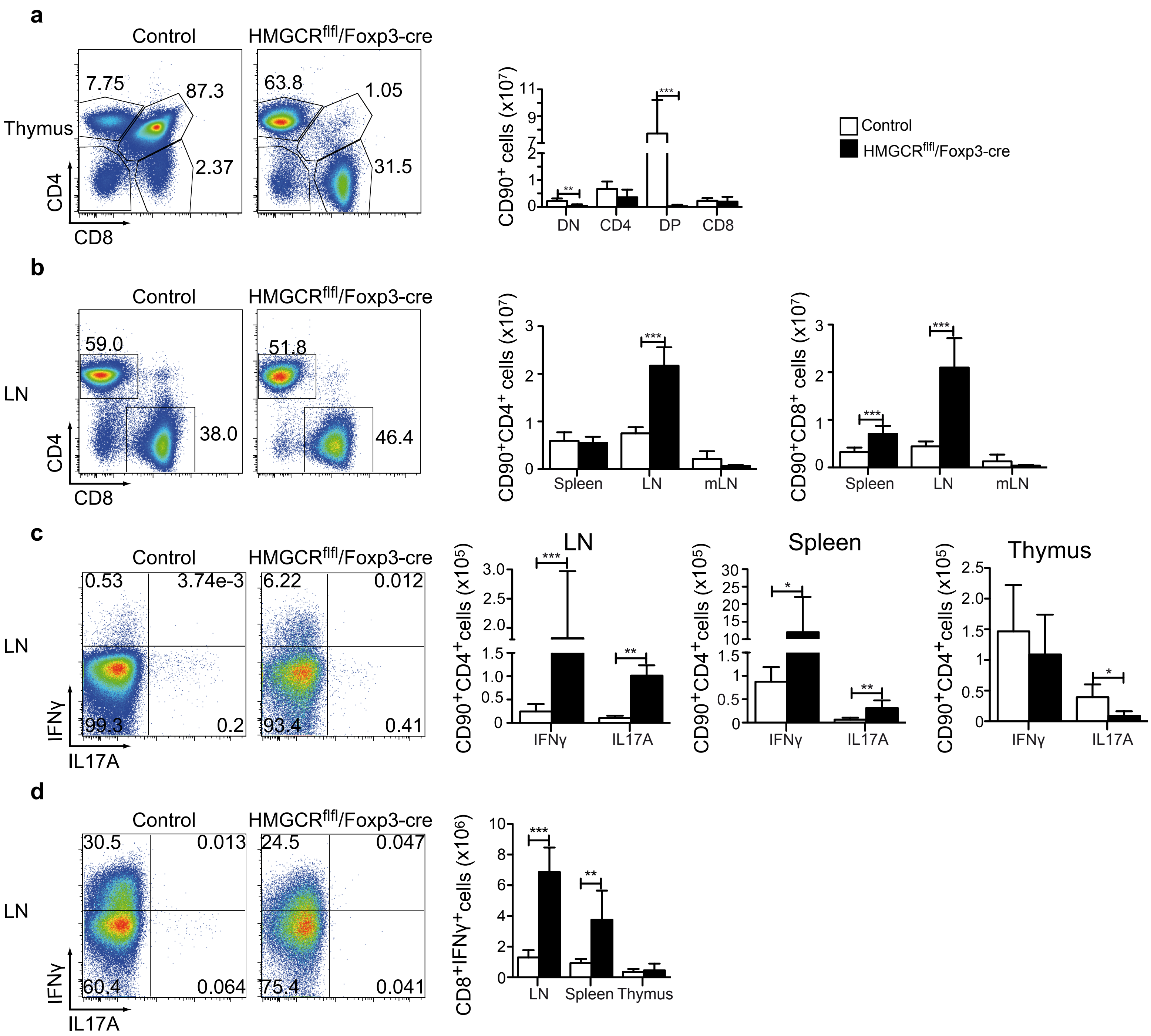

Supplementary Figure 5
