# Supplementary material for: HMG-CoA reductase promotes protein prenylation and therefore is indispensible for T-cell survival
Source: Cell Death Dis. 2017 May 25;8(5):e2824–. doi: 10.1038/cddis.2017.221 (PMC5520735; doi:10.1038/cddis.2017.221)
Supplement: Supplementary Figure Legend [file cddis2017221x2.docx]

**Supplementary Figure 1** Generation of the conditional HMGCR^flfl^ mice. Shown are schematic representations of the targeting vector (a), the WT locus (b), the locus after homologous recombination (c) and after the removal of the neomycin cassette by FLP recombinase, resulting in the HMGCR^flfl^ allele (d). Black boxes represent exons, separating lines represent introns. Also indicated are the Southern blot probes and lengths of constructs after EcoRI digestion in the Southern blot, shown in (e). The expected sizes of constructs are shown in the Southern blot for WT (+/+), HMGCR^het^ (+/-) and HMGCR^flfl^ mice (-/-).

**Supplementary Figure 2** HMGCR^het^/CD4-cre mice show no difference in EAE disease progression compared to controls. HMGCR^het^/CD4-cre mice (n=5+/-SEM) and the two control groups WT/CD4-cre (n=5+/-SEM) and HMGCR^het^ (n=5+/-SEM) were immunized with MOG/CFA and pertussis toxin and scored according to paralysis signs (a) or weight (b). The percentage of weight loss is indicated in (c).

**Supplementary Figure 3** Remaining T cells after HMGCR deletion are mostly activated. HMGCR^het^/CD4-cre control and HMGCR^flfl^/CD4-cre animals were further crossed to the YFP-reporter mice. Shown are YFP^+^CD4^+^ (a) and YFP^+^CD8^+^ (b) T cells. YFP^+^CD4^+^ T cells were further analyzed for percentage (c) and total cell numbers of regulatory CD25^+^Foxp3^+^ T cells (d) (n=5+/-SD).

**Supplementary Figure 4** HMGCR deletion results in the death of CD4^+^ (a) and CD8^+^ (b) T cells. VCT labeled splenocytes of HMGCR^flfl^/Rosa-cre^ERT2^ mice containing the YFP-Reporter were cultured under T cell activating conditions and 1µM 4-OH TAM for three days +/-Q-VD-OPh. Early apoptotic cells are gated as AnnexinV^+^7AAD^-^, late apoptotic cells as AnnexinV^+^7AAD^+^. As controls HMGCR^het^/Rosa-cre^ERT2^ animals with YFP-reporter were used (n=3+/-SD).

**Supplementary Figure 5** T_reg_-specific HMGCR deletion leads to a scurfy-like phenotype. Shown are CD90^+^ cells in thymus (a) or LN, spleen and mLN (b). Further the cytokine production by CD4^+^ (c) and CD8^+^ T cells (d) are shown; controls are: WT, Foxp3^+/Y^, HMGCR^het^/Foxp3-cre^+/-^, 2 HMGCR^het^/cre^+/+^, HMGCR^flfl^/Foxp3^+/-^; knockout HMGCR^flfl^/Foxp3-cre mice are HMGCR^flfl^/Foxp3^+/Y^ or HMGCR^flfl^/Foxp3^+/+^ (n≥5+/-SD).
